# Oxidative stress induced by hydrogen peroxide disrupts zebrafish visual development by altering apoptosis, antioxidant and estrogen related genes

**DOI:** 10.1038/s41598-024-64933-5

**Published:** 2024-06-24

**Authors:** Febriyansyah Saputra, Mitsuyo Kishida, Shao-Yang Hu

**Affiliations:** 1 Graduate School of Science and Technology, Kumamoto University, Kumamoto, Japan; 2 Department of Biological Science and Technology, National Pingtung University of Science and Technology, Pingtung, Taiwan

**Keywords:** Hydrogen peroxide, Oxidative stress, Visual development, Estrogen, Zebrafish, Developmental biology, Molecular biology, Apoptosis

## Abstract

Hydrogen peroxide is considered deleterious molecule that cause cellular damage integrity and function. Its key redox signaling molecule in oxidative stress and exerts toxicity on a wide range of organisms. Thus, to understand whether oxidative stress alters visual development, zebrafish embryos were exposed to H_2_O_2_ at concentration of 0.02 to 62.5 mM for 7 days. Eye to body length ratio (EBR) and apoptosis in retina at 48 hpf, and optomotor response (OMR) at 7 dpf were all measured. To investigate whether hydrogen peroxide-induced effects were mediated by oxidative stress, embryos were co-incubated with the antioxidant, glutathione (GSH) at 50 μM. Results revealed that concentrations of H_2_O_2_ at or above 0.1 mM induced developmental toxicity, leading to increased mortality and hatching delay. Furthermore, exposure to 0.1 mM H_2_O_2_ decreased EBR at 48 hpf and impaired OMR visual behavior at 7 dpf. Additionally, exposure increased the area of apoptotic cells in the retina at 48 hpf. The addition of GSH reversed the effects of H_2_O_2_, suggesting the involvement of oxidative stress. H_2_O_2_ decreased the expression of eye development-related genes, *pax6α* and *pax6β*. The expression of apoptosis-related genes, *tp53, casp3* and *bax*, significantly increased, while *bcl2α* expression decreased. Antioxidant-related genes *sod1*, *cat* and *gpx1a* showed decreased expression. Expression levels of estrogen receptors (ERs) (*esr1, esr2α*, and *esr2β*) and ovarian and brain aromatase genes (*cyp19a1a* and *cyp19a1b*, respectively) were also significantly reduced. Interestingly, co-incubation of GSH effectivity reversed the impact of H_2_O_2_ on most parameters. Overall, these results demonstrate that H_2_O_2_ induces adverse effects on visual development via oxidative stress, which leads to alter apoptosis, diminished antioxidant defenses and reduced estrogen production.

## Introduction

There is a growing recognition that hydrogen peroxide (H_2_O_2_) is also involved in genuine physiological processes^1^. This compound is notable as a highly stable and widespread reactive oxygen species (ROS) in aquatic ecosystems, arising not only as an unintentional toxic byproduct of metabolic processes within aquatic organisms but also from photochemical reactions occurring in natural water environments^2,3^. Additionally, atmospheric wet deposition has been identified as another significant source of H_2_O_2_ in aquatic ecosystems^4,5^. Aquatic environments may directly accumulate H_2_O_2_ due to anthropogenic activities like aquaculture, soil remediation, and wastewater treatment^6,7^. The concentration of H_2_O_2_ in natural water ranges widely, from 0.004 to 199 μM. These diverse concentrations have been extensively documented across various aquatic environments such as rivers (0.09–3.2 μM)^8^, lakes (0.00–5.31 μM)^9^, estuaries (0.01–0.35 μM)^10^, open oceans (0.06–0.45 μM)^11^, and rain (0–199 μM)^12^. High concentrations of H_2_O_2_ in aquatic environments can have detrimental effects on aquatic organisms, leading to abnormal physiological responses, altered behaviors, and compromised immune systems in species such as rainbow trout (*Oncorhynchus mykiss*)^13^, sea bass (*Dicentrarchus labrax*)^14^, sea bream (*Sparus aurata*)^15^. This highlights the critical need for further research and monitoring to understand and mitigate the potential negative effects of H_2_O_2_ levels on aquatic ecosystem.

H_2_O_2_ acting as a cell damaging agent that is produced during normal cellular metabolism, particularly during processes such as oxidative phosphorylation in mitochondria of aerobic organisms^16^. This includes a wide range of organisms, from microbes to plants and animals, all of which are capable of generating H_2_O_2_ as part of their regular cellular functions^17^. However, the excessive production of oxygen metabolites, including H_2_O_2_, results in oxidative stress and diseases^18^. Interestingly, recent research has unveiled a new perspective on H_2_O_2_. Despite its reputation as a harmful oxidant, H_2_O_2_ has been found to serve as a crucial signaling molecule with beneficial effects in specific cellular contexts^16^**.** Oxidative stress arises when oxidizing agents, free radicals, and reactive oxygen species surpass cellular antioxidant capacity^19^. ROS, byproducts of oxygen metabolism, play essential roles in physiological functions, including cell signaling. However, environmental stressors (such as UV radiation, ionising radiations, pollutants, and heavy metals) and xenobiotics (such as anticancer medications) significantly elevate ROS production, causing imbalance and cellular damage, also called oxidative stress^20^. Elevated ROS production induces adverse effects on crucial cellular structures, including proteins, lipids, and nucleic acids^21^. Evidence suggests oxidative stress's involvement in various diseases, including cancer, diabetes, metabolic disorders, atherosclerosis, endocrine dysfunction, infertility, neurological, and cardiovascular diseases^22–24^.

Increased ROS levels, coupled with normal antioxidant levels, induce oxidative stress in the brain, triggering apoptosis, cellular damage, and cell death. Elevated cell death often correlates with severe developmental abnormalities. Apoptosis, a common type of programmed cell death^25^, involves two main pathways in mammals and zebrafish: the intrinsic (also known as the mitochondrial pathway) and the cell-extrinsic pathway (death receptor pathway)^26^. The intrinsic pathway responds to various cellular stresses, including oxidative stress, and is regulated by pro-apoptotic genes like t*p53*^27^ and *bax*^28^, along with the suppression of anti-apoptotic members of the *bcl-2* gene family. It can be activated by numerous triggers including growth factor withdrawal and cellular damage^29^. Conversely, the extrinsic pathway begins with death receptor binding on the cell surface, initiating apoptosis through a death-inducing signalling complex. Both pathways ultimately converge on a shared group of effector caspases, such as caspase-3, which execute programmed cell death^30^.

Antioxidants protect organisms against the adverse impacts of free radicals, preventing or repairing damage. Superoxide dismutase (SOD) stands out among enzymatic antioxidants, scavenging superoxide radicals (O_2_^•−^) by converting them into less harmful molecules^31^. Various isoforms of SOD are present in distinct cellular compartments. SOD1 (CuZnSOD) serves as the primary superoxide scavenger in the cytoplasm, mitochondrial intermembrane spaces, nuclei, and lysosomes, while SOD2 (MnSOD) is located within the mitochondria^32^. Catalase, another crucial enzyme in intracellular defense against oxidative stress, is predominantly located in peroxisomes and cardiac mitochondria. It plays a vital role in breaking down H_2_O_2_ into water and oxygen, thus preventing its accumulation^33^. Additionally, glutathione peroxidases (GPXs) and peroxiredoxins (PRDXs) contribute to intracellular defenses against H_2_O_2_. These enzymes catalyze the reduction of H_2_O_2_ using reduced glutathione (GSH) as a cofactor, thereby detoxifying it and protecting cells from oxidative damage^34^. Hence, the coordinated action of these intracellular defense mechanisms helps to maintain cellular redox neutralisation and the protection of cells against oxidative stress-induced damage, which is implicated in various diseases.

For years, estrogen (E2) has been primarily produced in the gonads, occasionally in the adrenal cortex, under brain signaling. Estradiol, or E2, the primary type of estrogen, forms when testosterone is converted by aromatase, mainly in the ovaries and other glands. E2 enters the bloodstream, known for its reproductive role as a gonadal sex hormone^35^. However, it has become evident that the synthesis and impacts of E2 extend beyond reproductive tissues. E2 is synthesized locally through aromatase in various non-reproductive tissues and across the nervous system, induces significant, diverse effects on the development, maturation, and function within the nervous system^36^. Indeed, E2 has neuroprotective properties in the retina, protecting against excitotoxic cell death and retinal damage in humans. Furthermore, E2 influences eye anatomy, function, and the development of numerous ocular conditions^37^. Alteration in E2 levels caused by ageing or hormone therapy are linked to neurodegenerative retinal disorders and visual impairments^38^. Interference with estrogenic whether through medical interventions or exposure to environmental endocrine-disrupting compounds (EDCs), can directly or indirectly impact the visual system. Early exposure to these disruptors during development may result in long-term adverse effects^39^.

To achieve this, the zebrafish has been considered a suitable vertebrate animal model to study oxidative stress dynamics^40^ and visual neuroscience^41^. Due to its genetic conservation and similarity in retinal structure with other vertebrate species, the zebrafish is an ideal model for studying the human visual system^42^.^.^ The zebrafish, a prolific breeder, lays transparent embryos that remain see-through until 7 days post-fertilization (dpf), enabling real-time observation of neurogenesis. Consequently, the early development of the zebrafish visual system, including the retina, is extensively documented^43^. The zebrafish serves as a model system offering numerous advantages for studying cellular and genetic processes throughout vertebrate development and disease^44^.

In this study, our objective was to investigate the impact of H_2_O_2_ exposure on zebrafish visual development system, encompassing both morphological and molecular parameters. Through our research, we sought to elucidate whether these effects are primarily mediated by oxidative stress, aiming to gain deeper insights into the underlying mechanisms involved in these processes.

## Materials and methods

### Maintenance of zebrafish

Zebrafish wildtypes (*Danio rerio*) were obtained from Academia Sinica and housed in 40-L tanks at a constant temperature of 28 °C, following a light cycle of 14 h of light and 10 h of darkness. They were fed a standard diet of commercial pellets daily and supplemented with brine shrimp twice a day at 9:00 am and 5:00 pm. To facilitate mating, adult wild-type females and males were placed in a mating box at a ratio of 2:1 and separated by a partition to acclimate to their environment. The following day at 9:00 am, the partition was removed, allowing the zebrafish to mate under light stimulation. Eggs were harvested 30 min post-spawning, subjected to washing in circulating system water to remove dead embryos and impurities. Fertilized eggs were then rinsed in embryo medium (EM) containing 0.004% CaCl2, 0.163% MgSO4, 0.1% NaCl, and 0.003% KCl. Subsequently, they were evenly distributed into 6-well plates, with 30 embryos placed in each well containing 8 mL of EM, and maintained at a constant temperature of 28 °C. All procedures involving zebrafish were conducted in accordance with local animal welfare regulations and approved by the Institutional Animal Care and Use Committee (IACUC) of NPUST (approval No. NPUST-105–067).

### Exposure experiments

H_2_O_2_ and glutathione (GSH) were obtained from Sigma Aldrich and prepared as stock solutions at concentrations of 100 mM and 10 mM, respectively, in distilled water. These stock solutions were then diluted with embryo medium (EM) to achieve the desired concentrations of H_2_O_2_ (0.02, 0.1, 0.5, 2.5, 12.5, and 62.5 mM) for the experiment. Zebrafish embryos were exposed from 2 h post-fertilization (hpf) to 7 days post-fertilization (dpf) in 6-well plates, with 30 embryos in each well and three replicates per group. The exposed embryos were maintained at 28 °C, with exposure solutions changed daily. Dead embryos were removed immediately and zebrafish survival and hatching rates were observed every 24 h.

### Eye and body length measurement

Zebrafish embryos at 48 hpf were used for eye to body length measurement. At 48 hpf, the zebrafish eye undergoes crucial development, forming key structures such as the lens, retina, and optic nerve. This stage is vital for early eye development and the detection of abnormalities^45^. According to published protocol^46^, the entire eye diameter was assessed as the distance between the pigmented epithelium of one pole to the opposite pole, aligned with the spine. Body length was measured from the snout tip to the end of the spine before the caudal. The eye-to-body length ratio was assessed under a microscope (Leica 58APO) from the lateral view, and the data were converted into millimeters (mm). Each group was examined with ten embryos, and the experiments were replicated three times using eggs obtained from distinct spawns.

### Apoptosis cell assay

Apoptosis in the retina was conducted by using acridine orange (AO) staining at 48 hpf. Embryos were immersed in 5 µg/ml acridine orange (acridinium chloride hemi-[zinc chloride], Sigma Aldrich) in EM for 30 min and kept in the dark at 28 °C. Subsequently, embryos were rinsed repeatedly with EM, anesthetized with 0.016% tricaine, and positioned laterally before being mounted on a slide glass with 0.5% gel agarose. Apoptosis was examined using a fluorescent microscope (Leica M165 FC), with a focus on the eyes. The areas of apoptosis cell with fluorescent AO positivity were quantified using ImageJ software. Each group was examined with ten embryos, and the experiments were replicated three times using eggs obtained from distinct spawns.

### Visual behavior assay

**Figure 1:**
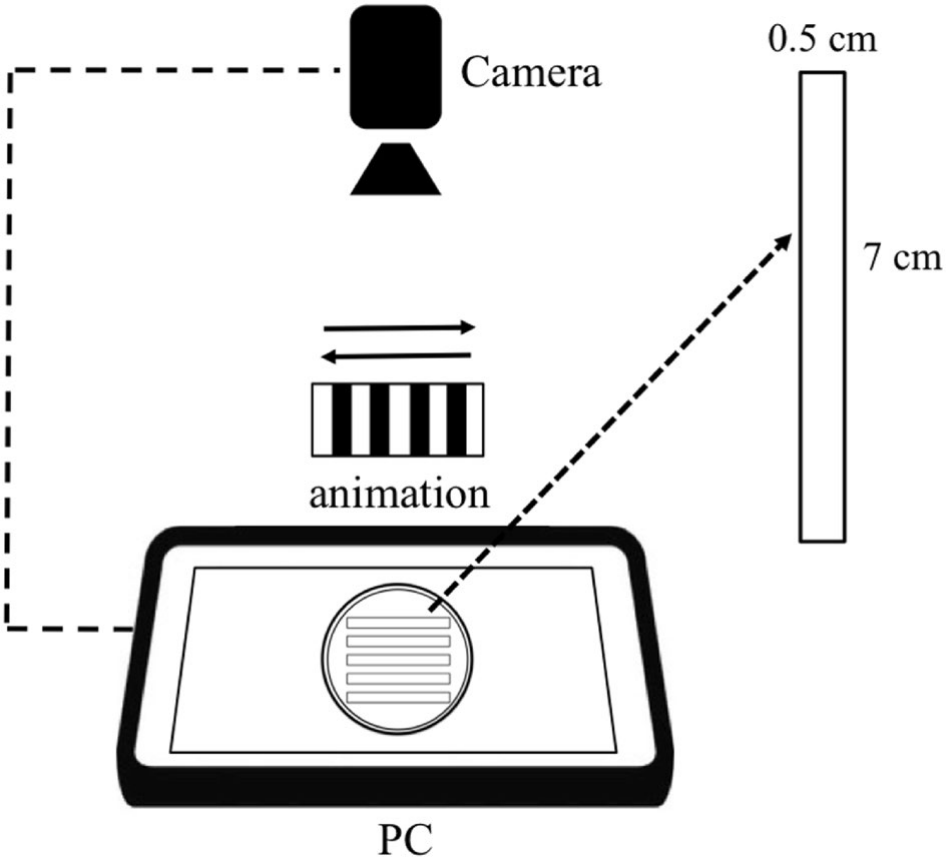
Apparatus for visual behavior assessment of OMR. Arrows (→) refers to the direction of animation. The experiments were conducted in the dark place at room temperature.

The optomotor response (OMR) was conducted for the visual behavior, which involves the movement of the head or body. OMR is effective in detecting abnormalities in visual function^47^. At 7 dpf, zebrafish larvae have developed enough to perform coordinated swimming movements, which makes it a suitable time point to assess motor activity and behavioral responses^48^. This method was adapted from a previously published protocol^49^. Zebrafish at 7 dpf were positioned in a specially designed petri dish containing five tracks (0.5 cm × 7 cm per track; Fig. 1) and were then incubated on a white screen for 30 s with light intensity at 825 nm. A study revealed that the visual behavior observed in zebrafish assays was responsive to light wavelengths spanning from 825 to 910 nm^50^. During the test, a white and black bars animation was presented moving both upward and downward at the same set of fixed speeds as a stimulus for the zebrafish, and the response was recorded for 30 s before and after measurement. All individuals moving in the following direction of the stimulus during the measurement are considered to demonstrate a positive OMR. The response of the zebrafish following the animation as indicated as positive OMR then measured in terms of swimming distance. Each group was examined with ten embryos, and the experiments were replicated three times using eggs obtained from different spawns.

### Real-time PCR

Zebrafish embryos were exposed to H_2_O_2_, and total RNA was extracted at 48 hpf (n = 20 per treatment group). Quantitative PCR was utilized to assess the expression levels of pair box protein (*pax6α* and *pax6β*), tumor protein 53 (*tp53*), caspase 3 (*casp3*), BCL2 associated X, apoptosis regulator a *(bax*), b-cell leukemia/lymphoma 2α (*bcl2α*), superoxide dismutase-1 (*sod1*), catalase (*cat*), glutathione peroxidase 1a (*gpx1a*), estrogen receptor (ERs) (*esr1, esr2α*, and *esr2β*), ovarian and brain aromatases (*cyp19a1a* and *cyp19a1b*, respectively), and elongation factor 1 alpha 1 (*eef1a1*) were determined using quantitative PCR. The *eef1a1* was used as an internal control. The specific primer used in this experiment are detailed in Table 1. Real-time PCR was carried out using KAPA SYBR FAST PCR reagent and an Applied Biosystems StepOnePlus Real-Time PCR system. The cycling profile included enzyme activation at 95 °C for 3 min, denaturation at 95 °C for 3 s, followed by annealing/primer extension for 40 cycles with denaturation at 95 °C for 3 s.

**Table 1:** Primer sequences.

Gene name	Primer sequence (5’-3’)	Accession number
*pax6α*	F: CTCAAACAGAAGAGCGAAATGGA	XM_009297889.3
	R: GAAGCTGCTGCTGATGGGTAT	
*pax6β*	F: CCTCCAGTCACATTCCCATCA	NM_131641.1
	R: AGCATTGAGCCTGTCGTGAA	
*tp53*	F: GGGCAATCAGCGAGCAAA	NM_131327.2
	R: ACTGACCTTCCTGAGTCTCCA	
*casp3*	F: CCGCTGCCCATCACTA	NM_131877.3
	R: ATCCTTTCACGACCATCT	
*bax*	F: CCGTGAGATCTTCTCTGATGG	NM_131562.2
	R: GTCAGGAACCCTGGTTGAAA	
*bcl2α*	F: AGGAAAATGGAGGTTGGGATG	NM_001030253.2
	R: TGTTAGGTATGAAAACGGGTGGA	
*sod1*	F: GTCGTCTGGCTTGTGGAGTG	NM_131294.1
	R: TGTCAGCGGGCTAGTGCTT	
*cat*	F: TACCAGTCAACTGCCCGTAC	NM_130912.2
	R: GACTCAAGGAAGCGTGGC	
*gpx1α*	F: GGCACAACAGTCAGGGATTA	NM_001007281.2
	R: CAGGACGGACGTATTTCAGA	
*esr1*	F: CCGGCCCTACACAGAGATCA	NM_152959.1
	R: AGCCAAGAGCTCTCCAACAACT	
*esr2α*	F: CTGTGCCGTCTGCAGTGATT	NM_180966.2
	R: CGGCGGTTCTTGTCGATAGT	
*esr2β*	F: TCCGACACCTCAGCAACAAA	NM_174862.3
	R: TTTCTGGGCTCTGTTGTCTGTCT	
*cyp19a1a*	F: AGATGTCGAGTTAAAGATCC	NM_131154.3
	R: ACTCGTTGATAAAACTCTCC	
*cyp19a1b*	F: GCAAATCGTACAGGAGATAC	NM_131642.2
	R: CGTCCAATGTTCAGGATTAG	
*eef1a1*	F: TGGTGGTGTCGGTGAGTTTG R: AAACGAGCCTGGCTGTAAGG	AY422992.1

### Ethics approval

The protocols for fish experiments were implemented according to local animal welfare regulations and approved (approval No. NPUST-105–067) by the Institutional Animal Care and Use Committee (IACUC) of NPUST. The study is reported in accordance with ARRIVE guidelines (https://arriveguidelines.org).

### Statistical analysis

Statistical analysis of all data was conducted through one-way ANOVA, followed by Tukey's post hoc test, utilizing the SigmaPlot 12.5 package for Windows. A significance level of *P* < 0.05 was applied in all analyses to identify significant differences between the treatments.

## Results

### Effects of H_2_O_2_ on survival and hatching rate

**Figure 2:**
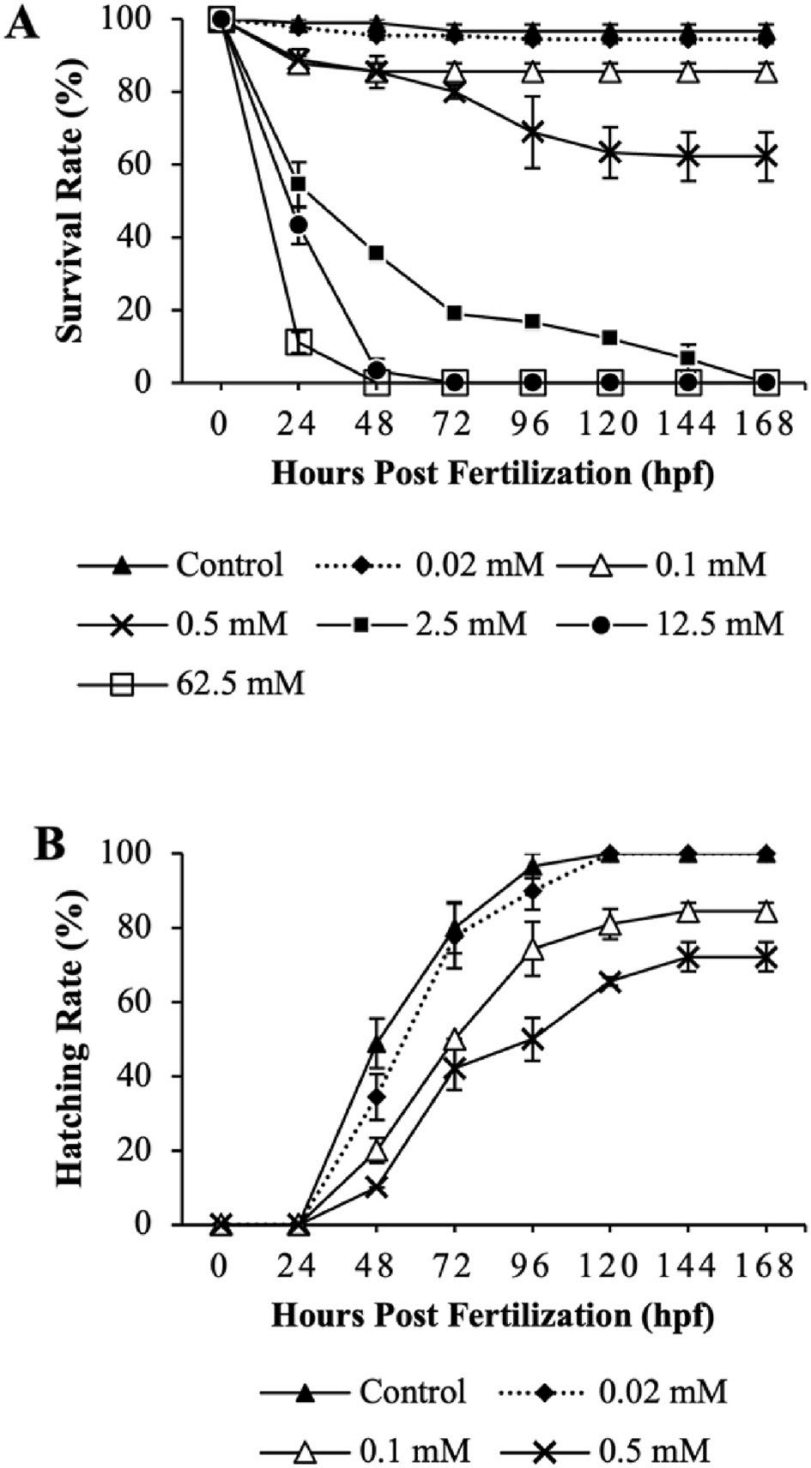
Survival (**A**) and hatching rate (**B**) of zebrafish embryos were measured following exposure to various concentration of H_2_O_2_ for 168 hpf. Each value is expressed as the mean ± SD of three replicates.

The study investigated the impact of different concentrations of H_2_O_2_ on the survival of zebrafish embryos and larvae over time, as depicted in Fig. 2A. While 0.02 mM showed no toxicity, 0.1 mM and 0.5 mM H_2_O_2_ showed toxicity, resulting in mortality rates of 14.4% and 37.8%, respectively, at 168 hpf. Higher concentrations of H_2_O_2_ (2.5 mM, 12.5 mM, and 62.5 mM) resulted in 100% mortality, with survival declining rapidly from 24 to 168 hpf. Consequently, 0.02 mM, 0.1 mM, and 0.5 mM of H_2_O_2_ will be further examined for their impact on zebrafish visual development. These results indicate that zebrafish development is sensitive to H_2_O_2_ in a time- and dose-dependent manner.

The hatching rates of zebrafish embryos exposed to varying concentrations of H_2_O_2_ at different developmental stages are presented in Fig. 2B. The results indicated a dose-dependent effect of H_2_O_2_ on the hatching rate under laboratory conditions. Compared to the control group, 0.02 mM of H_2_O_2_ did not impact the hatching rate up to 168 hpf. However, 0.1 mM and 0.5 mM H_2_O_2_ significantly delayed embryo hatching and exhibited toxicity.

### Effects of H_2_O_2_ on the eye to body length ratio

**Figure 3:**
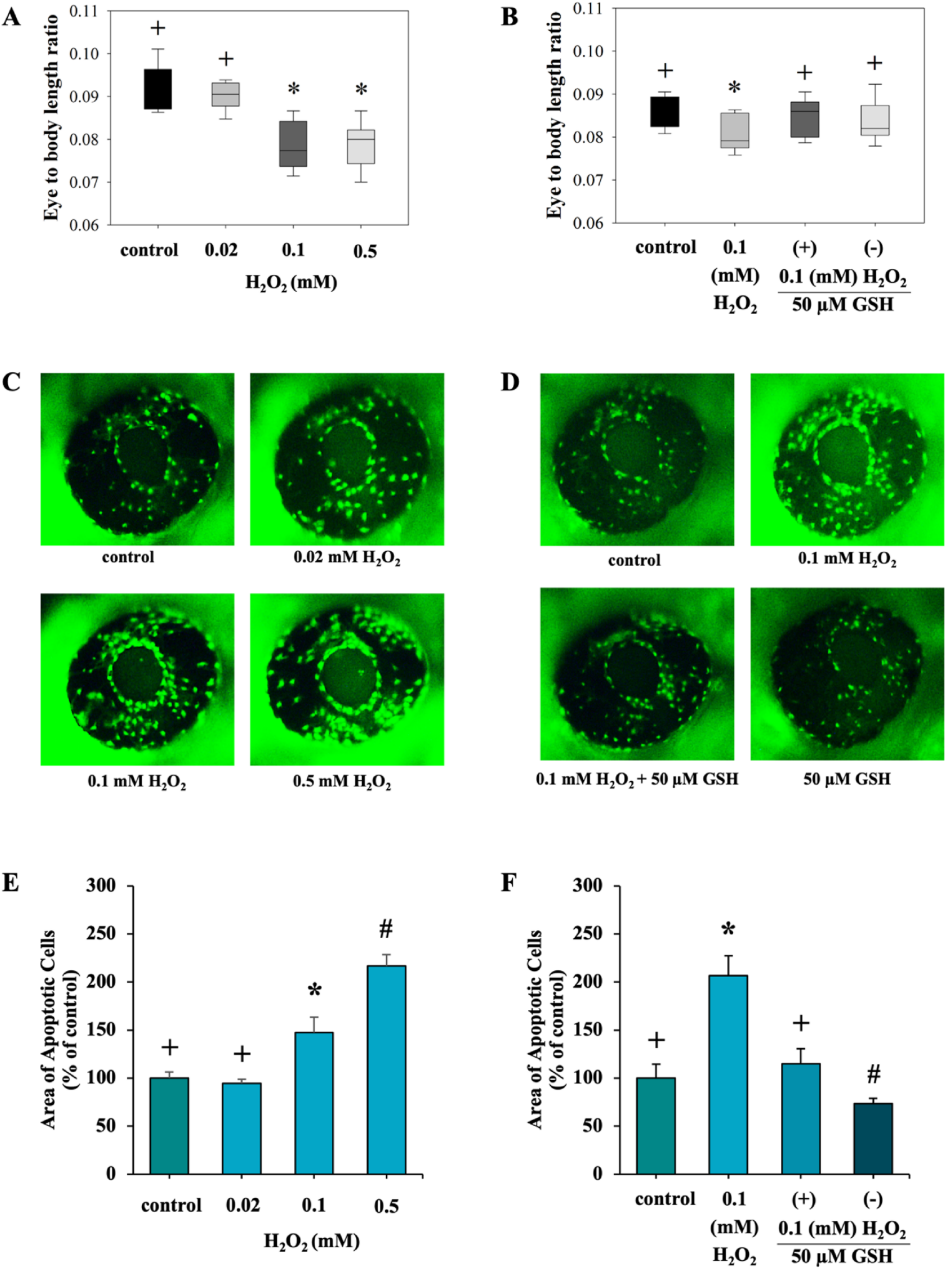
Effects of exposure to H_2_O_2_ and co-exposure to GSH on visual system. Eye to body length ratio was measured at 48 hpf: exposure to 0.02, 0.1, and 0.5 mM H_2_O_2_ (**A**), and co-exposure to 50 μM GSH with 0.1 mM H_2_O_2_ (**B**) (n = 10). Representative image of lateral views of apoptotic cells within the retina of live zebrafish embryos was observed at 48 hpf using AO staining: exposure to 0.02, 0.1, and 0.5 mM H_2_O_2_ (**C**), and co-exposure to 50 μM GSH with 0.1 mM H_2_O_2_ (**D**). Apoptosis signals were indicated by green fluorescent spotted (dot) on the retina. Area of apoptotic cells in the retina was measured at 48 hpf: exposure to 0.02, 0.1, and 0.5 mM H_2_O_2_ (**E**), and co-exposure to 50 μM GSH with 0.1 mM H_2_O_2_ (**F**) (n = 10). Each value is expressed as the mean ± SD. Different symbols in each graph indicate significant differences (p < 0.05). The experiments were repeated 3 times with different cohorts of eggs.

To determine whether H_2_O_2_ has a deleterious effect on zebrafish visual development, embryos were exposed to varying concentrations of H_2_O_2_ (0.02, 0.1, and 0.5 mM). As shown in Fig. 3A, exposure to 0.1 and 0.5 mM of H_2_O_2_ significantly decreased the eye-to-body length ratio at 48 hpf, Interestingly, the reduction induced by 0.1 mM of H_2_O_2_ was fully reversed upon the addition of GSH (Fig. 3B).

### Effects of H_2_O_2_ on the apoptosis in retina

An increase in fluorescence in the GFP channel correlates with increased AO staining and increased cell death in the embryo. Exposure to H_2_O_2_ at concentrations of 0.1 and 0.5 mM resulted in an increased the fluorescent AO positive cells (Fig. 3C) and area of apoptotic cells in the retina at 48 hpf (Fig. 3E). Notably, the augmentation induced by 0.1 mM of H_2_O_2_ was completely reversed upon the addition of GSH, as depicted in Fig. 3D,F.

### Effects of H_2_O_2_ on visual behavior

**Figure 4:**
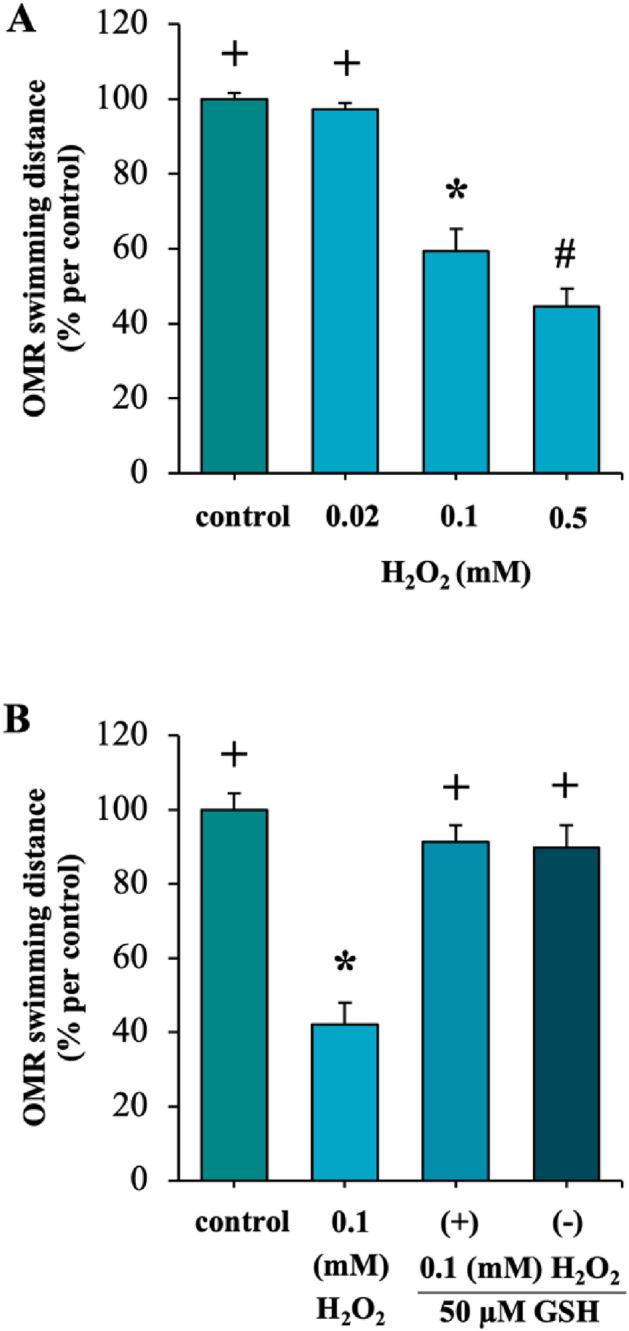
Effects of exposure to H_2_O_2_ and co-exposure to GSH on OMR assay. OMR was measured at 7 dpf: exposure to 0.02, 0.1, and 0.5 mM H_2_O_2_ (A), and co-exposure to 50 μM GSH with 0.1 mM H_2_O_2_ (B) (n = 10). Each value is expressed as the mean ± SD. Different symbols in each graph indicate significant differences (p < 0.05). The experiments were repeated 3 times with different cohorts of eggs.

Exposure to H_2_O_2_ affected zebrafish visual development at 48 hpf. To evaluate whether this change influenced visual behavior, OMR testing was conducted at 7 dpf. The results demonstrated a significant reduction in OMR swimming distance following exposure to 0.1 and 0.5 mM of H_2_O_2_, as depicted in Fig. 4A. The decrease induced by 0.1 mM of H_2_O_2_ was completely reversed by the addition of GSH (Fig. 4B).

### Effects of H_2_O_2_ on gene expression

**Figure 5:**
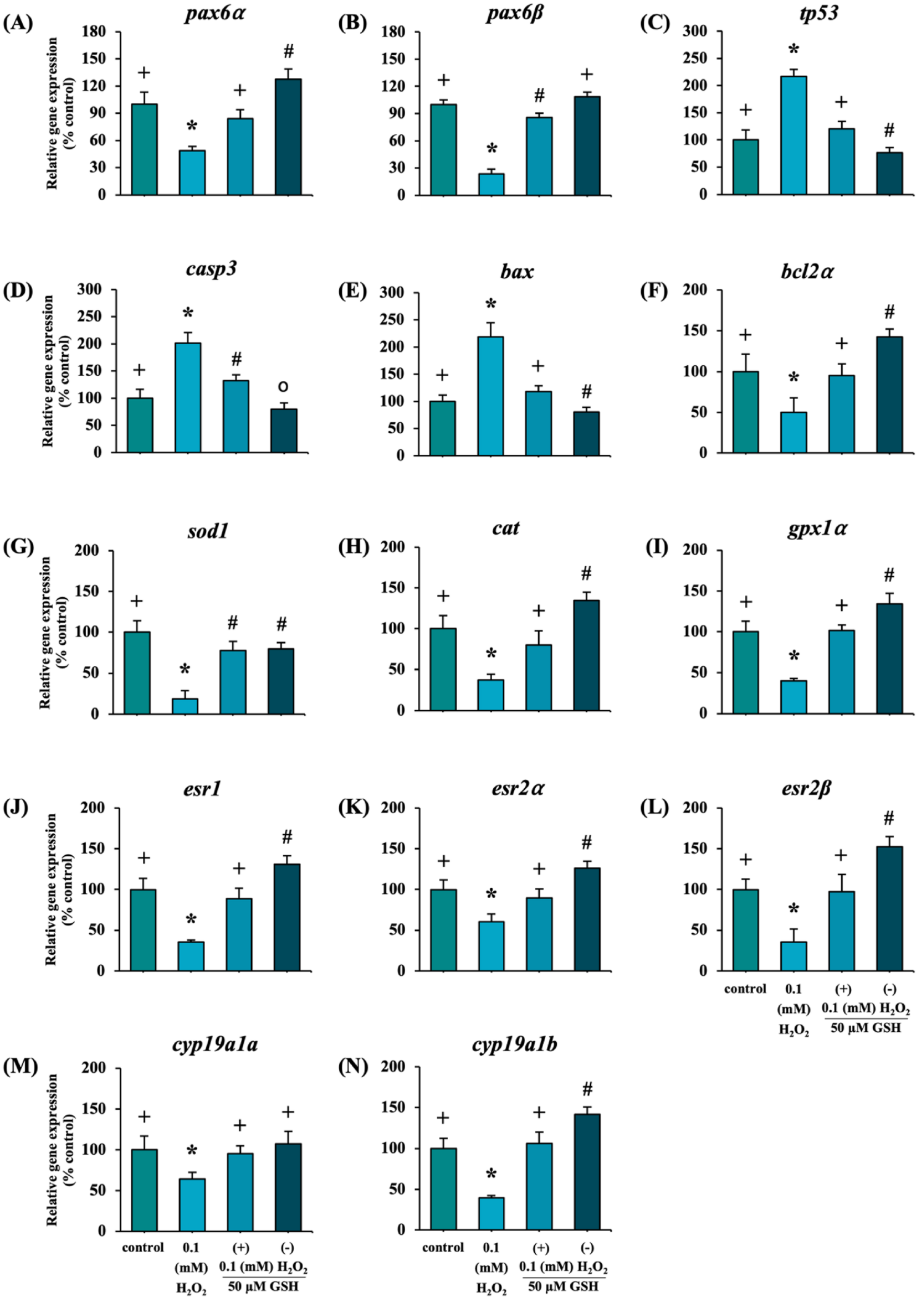
Effects of exposure to H_2_O_2_ and co-exposure to GSH on gene expression at 48 hpf measured by real-time PCR. Embryos were exposed to 0.1 mM H_2_O_2_ alone, or together with 50 μM GSH (n = 20 per treatment group). Relative expressions of genes related to eye development (**A** and **B**), apoptosis (**C**–**F**), antioxidant (**G** and **I**), ERs (**J**–**L**), aromatases (**M** and **N**). Each value is expressed as the mean ± SD. Different symbols in each graph indicate significant differences (p < 0.05). The experiments were repeated 3 times with different cohorts of eggs.

Exposure to H_2_O_2_ at 0.1 mM significantly decreased the relative expression of genes associated with visual development, *pax6α* and *pax6β* at 48 hpf, and these effects were significantly reversed by addition of GSH (Fig. 5A,B). The relative gene expression of *tp53*, *casp3* and *bax* significantly increased, while *bcl2a* was decreased, and these effects were also significantly reversed by the addition of GSH (Fig. 5C–F). As for antioxidant-related genes, exposure led to a significant decrease in the relative expression of *sod1*, *cat* and *gpx1α*, and these effects were significantly reversed by the addition of GSH (Fig. 5G–I). In terms of ERs, exposure to H_2_O_2_ significantly reduced the relative expression levels of *esr1*, *esr2α*, and *esr2β*, with the effects reversed upon GSH addition (Fig. 5J–L). Similarly, exposure resulted in a significant reduction in the relative expression levels of aromatase genes, *cyp19a1a* and *cyp19a1b*, which were also reversed by the addition of GSH (Fig. 5M,N).

## Discussion

Our study demonstrated the adverse impacts of H_2_O_2_ exposure on various aspects of zebrafish visual development. We observed detrimental effects on survival and hatching rate, eye to body length ratio, apoptotic cell in the retina, OMR swimming behaviour and genes related to eye development, apoptosis, antioxidant and estrogen signalling. Notably, our findings revealed a dose-dependent relationship, with significant effects observed starting from a concentration of 0.1 mM H_2_O_2_. Another study in common carp showed tissue-specific responses to H_2_O_2_ exposure, affecting immune, inflammatory, autophagic, and DNA damage pathways at 0.2 mM to 1 mM H_2_O_2_ concentrations^51^. Additionally, exposure to H_2_O_2_ concentrations ranging from 0 to 100,000 nM impacted the locomotion and metabolism of both zebrafish larvae and adults^52^. It's noteworthy that this concentration range falls within the spectrum of H_2_O_2_ levels found in aquatic environments such as lake, river, rain, estuary, and open ocean, ranging from 0.004 μM to 199 μM^9^. During the algal bloom period, levels exceeding 10,000 nM of H_2_O_2_ have been observed^53^. These results indicate the potential ecological relevance and implications of H_2_O_2_-induced toxicity in aquatic organisms like zebrafish.

To ascertain whether an eye is proportionally large relative to the subject's overall body size, eye to body length ratio were measured. Exposure to H_2_O_2_ resulted in a reduction of the eye-to-body length ratio in zebrafish at 48 hpf. This aligns with our observations, as previous research has utilized zebrafish to assess body length ratios, indicating that eye size is indeed proportionally correlation to overall body size^46^. Similarly, in other species, eye size has been shown to be tightly regulated relative to overall body size^54,55^, yet many animal models with eye defects exhibit abnormal body sizes^56^. Therefore, the decrease in eye size observed in zebrafish exposed to H_2_O_2_ may be correlated with a reduction in body length. This correlation suggests that the effects of H_2_O_2_ exposure extend beyond the visual system, potentially impacting overall growth and development in zebrafish. It's plausible that the oxidative stress induced by H_2_O_2_ could disrupt normal physiological processes, leading to alterations in both eye size and body length.

Apart from its significance as a biological phenomenon, the detection of apoptotic processes has been implicated in a wide range of diseases. Our research findings indicate that exposure to H_2_O_2_ resulted in an increase in the positive area of apoptotic cells in the retina of zebrafish at 48 hpf, as observed through AO staining. AO is a fluorescent dye binds to nucleic acids, specifically DNA and RNA, by intercalating between the base pairs and is utilized to investigate apoptosis in zebrafish and other model organisms. This property allows AO to stain DNA and RNA molecules, making them visible under a fluorescence microscope^57^. The observed increase in the positive area of AO fluorescence cells following exposure to H_2_O_2_ is correlated with elevated cell death. Thus, our study demonstrates that exposure to H_2_O_2_ leads to increased apoptotis in zebrafish retina at 48 hpf.

To investigate the impact of H_2_O_2_ exposure on visual behavior, we conducted the OMR assay. The OMR is an innate visuomotor reflex observed in zebrafish, where the fish aligns its swimming direction with a high-contrast visual stimulus to stabilize its position relative to the stimulus. This assay is commonly employed to assess changes in vision-related behaviors^58^. Utilizing the OMR task in zebrafish has proven effective in detecting visual abnormalities induced by genetic mutations and toxin exposure^59,60^. Our study revealed that exposure to H_2_O_2_ resulted in reduced swimming behavior in response to the positive directions of the OMR stimulus. This finding implies that defects caused by H_2_O_2_ exposure may contribute to abnormal visual behavior, suggesting a potential association between H_2_O_2_ exposure and visual impairments.

Exposure significantly altered relative genes expression involved in eye development, apoptosis, antioxidant and estrogen signalling were assessed by qPCR at 48 hpf. *Pax6* is essential for the development of the eye in numerous species, including flies, zebrafish, mice, and humans^61^. Furthermore, *pax6* is expressed in neuronal progenitor cells within the regeneration process of the adult zebrafish retina^62,63^. Zebrafish possess two paralogous *pax6* genes, *pax6α* and *pax6β*, which encode functionally redundant proteins responsible for regulating the formation and differentiation of the retina and lens^64^. The knockdown of either *pax6α* and *pax6β* resulted in the disruption of retinal regeneration in zebrafish^65^. Reduced expression of *pax6* has been associated with the loss of eye function^66^. Our results align with this finding, demonstrating a significant decrease in the expression of both *pax6α* and *pax6β* following exposure to H_2_O_2_. This suggests that the exposure may impair eye function, potentially contributing to the abnormalities observed in visual behavior as detected by the OMR assay.

Our study demonstrated that exposure to H_2_O_2_ elevated cell death in the retina at 48 hpf. The *tp53* gene is a well-known tumor suppressor gene responsible for regulating both cell proliferation and cell death in response to DNA damage^67^. Various DNA-damaging agents have been demonstrated to elevate zebrafish *tp53* transcription^68^ and enhance *tp53* protein levels^69^. There is substantial evidence indicating that death signals triggered by *tp53* lead to the activation of caspases. Multiple studies have been carried out to clarify the function of caspase activation in cell death mediated by *tp53*^70–73^. Transcripts for caspase-3 are present during early embryogenesis^74^. Additionally, caspase-3 is a key player in the advancement of apoptosis, resulting in changes in cell morphology and DNA fragmentation. Our study demonstrated that exposure to H_2_O_2_ increased *tp53* and *caps3*, indicating that cell death induced by H_2_O_2_ follows an apoptotic mechanism. This implies that the cellular response to H_2_O_2_ involves the activation of apoptotic pathways mediated by *tp53* and *caps3*, which play crucial roles in regulating programmed cell death. Moreover, mitochondria play a significant role in one of the primary pathways for caspase activation. *Bcl-2* suppresses apoptosis and enhances cell survival, while *bax* operates within the mitochondria to trigger the production of cytochrome c, initiating the caspases activation^75^. In the present study, H_2_O_2_ reduced *bcl2α* expression and increased *bax*, indicating the involvement of mitochondria in H_2_O_2_-induced apoptosis, leading to developmental defects in the visual system of zebrafish.

H_2_O_2_ has the capacity to initiate apoptosis via diverse mechanisms through generating oxidative stress^76^. Oxidative stress arises from an imbalance between ROS production and the ability of the body's antioxidant defenses to neutralize them. In this study, H_2_O_2_ exposure induced alterations in antioxidant-related genes such as *sod1, cat,* and *gpx1a*. SOD1, part of the SOD family, crucial endogenous antioxidant enzymes that serve as the primary defense against ROS within cells. It catalyses the dismutation of superoxide into H_2_O_2_ and oxygen, thus reducing the harmful effects of superoxide anion. Catalase or glutathione peroxidase detoxify H_2_O_2_ and convert it into water. Catalase efficiently breaks down H_2_O_2_, while glutathione peroxidase also aids in converting H_2_O_2_ into water and oxygen. Glutathione peroxidases, particularly GPx1, have been associated with both the onset and prevention of various common and complex diseases^77^. Studies have shown that *gpx1* provide protection against apoptosis triggered by oxidative stress^78^, while also decreasing the expression *bax* (pro-apoptotic protein)^79^. We demonstrated that H_2_O_2_ reduced the expression of *sod1, cat,* and *gpx1a*, while increased the pro-apoptotic gene. This suggests a decrease in antioxidant capacity and an increased vulnerability to oxidative stress, which could trigger apoptosis, or programmed cell death, potentially contributing to visual dysfunction during zebrafish development.

Oxidative stress plays a significant role in influencing E2 signaling pathways by modulating the activity of estrogen receptors (ERs)^80^. E2, crucial for various physiological functions, is synthesized from testosterone through the action of the aromatase enzyme^81^. In zebrafish, two distinct aromatase genes are present: *cyp19a1a*, which encodes ovarian aromatase found in the gonads, and *cyp19a1b*, which encodes brain aromatase found in neural tissues such as the brain and retina^36,82^. The physiological effects of estrogen primarily occur through its binding to two types of ERs. Zebrafish possess two types of ERs, namely *Erα* and *Erβ*, which are encoded by three distinct genes: *esr1*, *esr2α*, and *esr2β*^83^. While E2 might possess an essential role in maintaining eye health, the exact mechanisms are still not fully understood. Nevertheless, hormone replacement therapies have been linked to lower rates of eye diseases like glaucoma, AMD, and cataracts, indicating that estrogen could be important for eye health^84^. The impact of oxidative stress induced by H_2_O_2_ exposure on the expression of ERs (*esr1, esr2α*, and *esr2β*) and aromatases (*cyp19a1a* and *cyp19a1b*) in zebrafish was examined. Our results demonstrated that H_2_O_2_ decreased the expression of ERs (*esr1, esr2α*, and *esr2β*) and aromatases (*cyp19a1a* and *cyp19a1b*), likely due to the influence of excessive oxidative stress production induced by H_2_O_2_. Previous studies have highlighted the pivotal role of E2 in eye development and function, as evidenced by the presence of aromatase in the retina and ERs distributed across various retinal layers in different vertebrate species^85,86^. The oxidative stress induced by H_2_O_2_ exposure may lead to decreased estrogen production, potentially impacting visual function in zebrafish, given the importance of estrogen for maintaining visual development. Therefore, understanding the impact of oxidative stress on estrogen-related pathways is critical for elucidating the mechanisms underlying visual development and potential implications for visual disorders.

Interestingly, co-incubation of GSH effectivity reversed the impact of H_2_O_2_ on most parameters, suggesting that oxidative stress mediates these effects. Glutathione serves as a vital antioxidant pivotal in mitigating oxidative stress within cellular environments. Its principal function involves the neutralization of ROS and shielding cells from oxidative damage^77^. This principal function of GSH involves the scavenging of ROS, including hydroxyl radicals (^•^OH), superoxide radicals (O_2_^•-^), and peroxyl radicals (ROO^•^), which are generated during oxidative stress conditions. By donating an electron to these highly reactive species, GSH effectively neutralizes them and prevents them from causing oxidative damage to cellular structures such as lipids, proteins, and DNA^87^. Prior research indicates that providing glutathione helps protect cells from oxidative stress-induced damage^88^. Conversely, reduced levels of glutathione, which lead to the impairment of the glutathione-dependent enzyme pathway, are linked to the development and advancement of numerous diseases^89^. Therefore, the addition of GSH unequivocally illustrated that the impact of H_2_O_2_ on zebrafish visual development is mediated through oxidative stress. In some cases, GSH alone alter genes expression, suggesting that antioxidants might affect gene regulation by modulating pathways associated with the response to oxidative stress. Numerous investigations have also demonstrated that bursts of oxidant generation, as well as significant variations in the responses of several antioxidant defences, are strongly related with changes in gene expression across a range of tissues from genetically distinct organisms^90,91^. GSH effectively mitigates oxidative stress and reduces the risk of cellular damage and apoptosis^77^. Therefore, the protective effects of GSH in zebrafish exposed to H_2_O_2_ are attributed to its dual role in free radical quenching and antioxidant activity, which collectively help maintain cellular integrity and promote survival under oxidative stress conditions.

## Conclusion

In summary, our study showed that exposure to H_2_O_2_ triggers oxidative stress, resulting in adverse effects on the visual development of zebrafish, including changes in eye-to-body length ratios, apoptotic activity in the retina, and altered OMR responses. These alterations are linked to the excessive oxidative stress, resulting in apoptosis, antioxidant imbalance, reduced estrogen production, and impaired zebrafish visual development. These findings demonstrate the critical role of oxidative stress in mediating adverse effects on visual function and development, emphasising the importance of antioxidant defenses in protecting against such harmful effects.

## Data Availability

Data will be available from the corresponding author upon reasonable request.
