# Author Correction: Oxidative stress induced by hydrogen peroxide disrupts zebrafish visual development by altering apoptosis, antioxidant and estrogen related genes

**DOI:** 10.1038/s41598-024-72932-9

**Published:** 2024-09-25

**Authors:** Febriyansyah Saputra, Mitsuyo Kishida, Shao‑Yang Hu

**Affiliations:** 1 Graduate School of Science and Technology, Kumamoto University, Kumamoto, Japan; 2 Department of Biological Science and Technology, National Pingtung University of Science and Technology, Pingtung, Taiwan

Correction to: *Scientific Reports*10.1038/s41598-024-64933-5, published online 24 June 2024

The original version of this Article contained errors, where the molecule H_2_O_2_ was misspelt.

As a result, in the Materials and methods section, under the subheading ‘Exposure experiments’,

“H_2_O^2^ and glutathione (GSH) were obtained from Sigma Aldrich and prepared as stock solutions at concentrations of 100 mM and 10 mM, respectively, in distilled water.”

now reads:

“H_2_O_2_ and glutathione (GSH) were obtained from Sigma Aldrich and prepared as stock solutions at concentrations of 100 mM and 10 mM, respectively, in distilled water.”

In addition, under the Results section, the subheading ‘Effects of H2O2 on survival and hatching rate’

now reads:

‘Effects of H_2_O_2_ on survival and hatching rate’

And under the subheading ‘Real-time PCR’

“Zebrafish embryos were exposed to nitrate and nitrite, and total RNA was extracted at 48 hpf (n = 20 per treatment group).”

now reads:

“Zebrafish embryos were exposed to H_2_O_2_, and total RNA was extracted at 48 hpf (n = 20 per treatment group).”

The original Article has been corrected.

